# Homœopathy in Public Hospitals

**Published:** 1882-02

**Authors:** 


### HOMŒOPATHY IN PUBLIC HOSPITALS.

The recent admission of homœopaths to the Chicago City Hospital is a welcome, though tardy acknowledgment of the merits of our system. It is to be hoped that, at no distant day, Homœopathy will gain a position in all our public institutions. But the profession in general, and especially those representing our school in these institutions, must remember that we can only excel our allopathic *brethren* by practicing Homœopathy in its purity. If those who are to represent Homœopathy in the Chicago Hospital fail to practice Homœopathy in its purity they will also fail to excel their rivals in practical results; and will, moreover, furnish their opponents with a grand weapon for use against the school they represent. The allopathic gentlemen do not look with friendly eye upon this admission of the homœopath to their before undisputed domains. They have doubtless read enough of homœopathic literature to know what kind of practice its law demands; and, we may be sure, they will be careful to note any lapse from strict adherence to this law. So that any resort to *morphia*, *quinia*, *cathartics*, etc., etc., will be met with the pertinent inquiry: Why introduce *homœopaths* into our hospitals to practice medicine as we are accustomed to practice it? What answer could homœopathists make to such inquiry? We by no means intend to assert that the gentlemen who have been appointed to the Chicago Hospital are so faithless to their better knowledge as to use these expedients; we merely desire to make plain the responsibility they assume in undertaking to represent Homœopathy in this hospital. The honor of our school and the success of each practitioner alike demand that they practice pure Homœopathy.

